# The Relationship Between Nutrition Habits, BMI, Anxiety, and Seborrheic Dermatitis

**DOI:** 10.1111/jocd.16737

**Published:** 2025-01-06

**Authors:** Tayfun Batan, Ersoy Acer, Hilal Kaya Erdoğan, Esra Ağaoğlu, Muzaffer Bilgin, Zeynep Nurhan Saraçoğlu

**Affiliations:** ^1^ Dermatology and Venereology Department Health Sciences University Beyhekim Education and Research Hospital Konya Turkey; ^2^ Dermatology and Venereology Department Eskisehir Osmangazi University Eskisehir Turkey; ^3^ Biostatistics Department Eskisehir Osmangazi University Eskisehir Turkey

**Keywords:** body mass index, nutrition, psychological aspects, seborrheic dermatitis

## Abstract

**Background:**

Seborrheic dermatitis (SD) is a chronic, inflammatory disease characterized by unknown etiopathogenesis. It affects skin areas rich in sebaceous glands. There are strong data on the relationship between nutrition habits, body mass index (BMI), psychoemotional status, and sebaceous gland diseases such as acne, rosacea, and androgenetic alopecia. However, there are very little data on SD, nutrition habits, BMI, and psychoemotional status.

**Aims:**

We aimed to evaluate the nutrition habits, BMI, and psychoemotional status in patients with SD.

**Methods:**

One hundred patients with SD and 110 healthy controls aged 18–65 years were included. Adolescents Food Habits Checklist (AFHC), a questionnaire form consisting information about nutrition habits and Depression Anxiety Stress Scale‐21 (DASS‐21) were completed by the participants, and BMI was calculated.

**Results:**

Severity of SD was positively correlated with BMI (*p* = 0.018). Patients with SD consumed more bread and less fruits–vegetables (*p* = 0.001, *p* = 0.006). Margarine, animal fat, and sugar consumption was higher in patients with moderate to severe SD (*p* = 0.008, *p* = 0.050). AFHC score was lower in patients with SD (*p* = 0.009). DASS‐21 anxiety subscale and DASS‐21 total scores were higher in the moderate to severe SD group (*p* = 0.035, *p* = 0.049).

**Conclusions:**

Nutrition habits, higher BMI, and psychoemotional status may play a critical role in the etiopathogenesis of SD. Healthy nutrition habits and psychoemotional status may prevent the occurrence and exacerbation of SD.

## 1 Introduction

Seborrheic dermatitis (SD) is a common, inflammatory, chronic, and recurring disease of skin regions with a high density of sebaceous glands [1, 2]. Personal and environmental factors such as *Malassezia* yeasts, sebaceous secretion, genetic predisposition, lipid composition of the skin, hormones, immune status, neuropsychiatric diseases, higher body fat content, and obesity have been blamed in the etiopathogenesis of SD, but it isn't entirely elucidated [1, 2, 3, 4, 5, 6, 7].

There are strong data on the relationship between nutrition habits, psychoemotional status, and the other sebaceous gland diseases such as acne, rosacea, and androgenetic alopecia [8, 9, 10]. However, there is limited knowledge on SD and nutrition. We aimed to evaluate the nutrition habits, body mass index (BMI), and psychoemotional status in patients with SD.

## 2 Materials and Methods

One hundred patients with SD and 110 healthy controls aged 18–65 years who consulted to Eskişehir Osmangazi University, Medical Faculty, Dermatology Outpatient Clinic between December 30, 2019 and December 30, 2020 were included in the study. SD was diagnosed by clinical assessment of erythema and oily scales in sebaceous gland–rich areas with a characteristic distribution. The control group consisted of those who applied to our outpatient clinic with diagnoses such as melanocytic nevus or verruca vulgaris and who did not have any systemic or psychodermatological disease and any medication. Demographic characteristics of control group were similar to the patient group. Subjects under 18 years old or over 65 years old, with any psychiatric illness, eating disorder, history of a special diet, inflammatory skin diseases, chronic systemic diseases, cognitively incapable of completing the questionnaires on their own, pregnant, or breastfeeding mothers were excluded.

The local ethics committee approved the study protocol (Decision no: 2020/16). All participants signed the informed consent form before enrollment. Dermatological examinations were performed, and sociodemographic information was recorded for the patients and volunteers. Participants were asked to fill self‐questionnaires including background, demographic data, and nutrition habits such as fat consumption preference, salt, and spice consumption, consumption frequency of tea–coffee, sugar, bread, vegetables–fruits, red meat, and milk. Additionally, Depression Anxiety Stress Scale‐21 (DASS‐21) and Adolescents Food Habits Checklist (AFHC) were fulfilled by the participants. Severity of disease was evaluated with the seborrheic dermatitis area severity index (SDASI).

AFHC was developed by Johnson et al. in 2002, and it has validity and reliability study in Turkey. Respondents were asked to give one of the following responses to the statements evaluating their healthy eating habits: “true, false or no idea/no practice”. High score is interpreted as healthy in terms of eating habits [11, 12].

The DASS‐21 scale was created from some propositions of the DASS‐42, and it has validity and reliability study in Turkey. The main function of the scale is to assess the severity of symptoms of depression, anxiety, and stress. It is a 4‐point Likert scale, coded as 0: “completely unsuitable for me”, 1: “somewhat suitable for me”, 2: “generally suitable for me”, and 3: “completely suitable for me”. The participants were asked to answer the scale according to his/her emotional state in the last week [13, 14].

The SDASI is an adaptation of the Psoriasis Area Severity Index used for psoriasis. It is a scoring system that includes different rates according to the areas of involvement and severity. Erythema and desquamation are graded in nine different anatomical regions. According to the degree of erythema and desquamation, it is scored as 0: absent, 1: mild, 2: moderate, and 3: severe. The score of erythema and desquamation in each anatomical region is multiplied by the constant for that region (forehead [0.1], scalp [0.4], nasolabial [0.1], eyebrow [0.1], postauricular [0.1], auricular [0.1], intermammary [0.2], back [0.2], and cheek or chin [0.1]). All regions are collected for the total score. The resulting score is called SDASI and ranges from 0 to 12.6 [15]. In this study, patients with ≥ 4 SDASI score and/or with ≥ 3 anatomical region involvement were also considered clinically moderate to severe SD and ≤ 3 SDASI score and/or with ≤ 2 anatomical region involvement had been considered mild SD [16].

### 2.1 Statistical Analysis

The data were analyzed with SPSS Statistics for Windows, version 21.0 (Armonk, NY: IBM Corp.) Continuous data were expressed as mean ± standard deviation. Categorical data were expressed as percentages (%). We conducted Shapiro–Wilk test to examine the suitability of the data for normal distribution. If the two groups were normally distributed, independent sample *t*‐test was done. However, groups were not normally distributed, Mann–Whitney *U* test was done. Pearson chi‐squared and Pearson exact chi‐squared were conducted to analyze the cross tables. Pearson correlation coefficients were computed for variables with normal distribution to define the direction and magnitude of the relationship (correlation) between variables. A value of *p* < 0.05 was considered statistically significant.

## 3 Results

### 3.1 Sociodemographic Characteristics

One hundred patients with SD and 110 healthy controls were included in the study. Of the patients with SD, 47% (*n* = 47) were female, and 53% (*n* = 53) were male; of the control group 53.6% (*n* = 59) were female, and 46.4% (*n* = 51) were male (*p* = 0.41). The mean age was 30.1 ± 11.5 years in patients with SD and 31 ± 8.79 years in the control group (*p* = 0.06). The mean BMI was 25 ± 4.28 in the patients with SD and 24.1 ± 3.78 in the control group (*p* = 0.22) (Table 1).

**TABLE 1:** Sociodemographic characteristics and nutrition habits in controls and patients with SD.

Variables	Controls (*n* = 110)	SD (*n* = 100)	*p*
Gender			
Male	51 (46.4%)	53 (53%)	0.41^a^
Female	59 (53.6%)	47 (47%)	
Age	31 ± 8.79	30.1 ± 11.5	0.06^b^
BMI	24.1 ± 3.78	25 ± 4.28	0.22^c^
AFCH	10.3 ± 3.97	8.92 ± 3.75	**0.009** ^c^
Diet preference			
Mostly meat	24 (21.8%)	17 (17.0%)	0.56^e^
Vegetarian	1 (0.9%)	2 (2.0%)	
Mixed	85 (77.3%)	81 (81.0%)	
Oil preference			
Vegetable oil	36 (32.7%)	25 (25.0%)	0.20^d^
Animal oil	6 (5.5%)	2 (2.0%)	
Margarine	0 (0%)	1 (1.0%)	
Mixed	68 (61.8%)	72 (72.0%)	
Salt consumption			
Less salty	39 (35.5%)	31 (31.0%)	0.27^a^
More salty	14 (12.7%)	21 (21.0%)	
Normal	57 (51.8%)	48 (48.0%)	
Spice consumption	30 (27.3%)	32 (32.0%)	0.73^d^
Less spicy	0 (0%)	0 (0%)	
More spicy	20 (18.2%)	18 (18.0%)	
Normal	60 (54.5%)	50 (50.0%)	
Tea–coffee consumption			
1–4/day	54 (49.1%)	47 (47.0%)	0.26^d^
5–6/day	35 (31.8%)	24 (24.0%)	
7–10/day	9 (8.2%)	15 (15.0%)	
≥ 10/day	8 (7.3%)	6 (6.0%)	
Never	4 (3.6%)	8 (8.0%)	
Sugar consumption			
1/month	7 (6.4%)	1 (1.0%)	0.13^d^
2/month	3 (2.7%)	1 (1.0%)	
1–2/week	26 (23.6%)	23 (23.0%)	
3–6/week	15 (13.6%)	13 (13.0%)	
Every day	26 (23.6%)	37 (37.0%)	
Never	33 (30.0%)	25 (25.0%)	
Bread consumption			
1/month	4 (3.6%)	3 (3.0%)	**0.001** ^**d**^
2/month	3 (2.7%)	1 (1.0%)	
1–2/week	31 (28.2%)	8 (8.0%)	
3–6/week	20 (18.2%)	16 (16.0%)	
Every day	42 (38.2%)	64 (64.0%)	
Never	10 (9.1%)	8 (8.0%)	
Milk consumption			
1/month	8 (7.3%)	9 (9.0%)	0.75^a^
2/month	10 (9.1%)	6 (6.0%)	
1–2/week	38 (34.5%)	40 (40.0%)	
3–6/week	11 (10.0%)	13 (13.0%)	
Every day	24 (21.8%)	16 (16.0%)	
Never	19 (17.3%)	16 (16.0%)	
Vegetable–fruit consumption			
1–2/week	46 (41.8%)	46 (46.0%)	**0.006** ^d^
1/day	41 (37.3%)	32 (32.0%)	
2/day	20 (18.2%)	13 (13.0%)	
≥ 3/day	0 (0%)	9 (9%)	
Never	3 (2.7%)	0 (0%)	
Red meat consumption			
1–2/week	87 (79.1%)	81 (81.0%)	0.052^d^
1/day	22 (20%)	12 (12.0%)	
2/day	0 (0%)	4 (4%)	
≥ 3/day	1 (0.9%)	3 (3.0%)	
Never	0 (0%)	0 (0%)	

*Note:* Bold value of *p* 〈 0.05 was considered statistically significant.

Abbreviations: AFCH, Adolescent Food Habits Checklist; BMI, body mass index, SD: seborrheic dermatitis.

^a^ Pearson chi‐squared.

^b^ Mann–Whitney *U* test.

^c^ Independent student *t*–test.

^d^ Pearson exact chi‐squared.

^e^ Fisher exact chi‐squared.

Patients with SD were classified as mild and moderate to severe according to SD severity. Of the total 100 patients, 79% (*n* = 79) had mild SD, and 21% (*n* = 21) had moderate to severe SD. Gender and age distribution was similar in the mild and moderate to severe patients (*p* = 0.5, *p* = 0.172, respectively). The mean BMI was 24.50 ± 4.15 in the mild group and 27.39 ± 4.76 in the moderate to severe group. The mean BMI was significantly higher in the moderate to severe patients (*p* = 0.01) (Table 2).

**TABLE 2:** Sociodemographic characteristics and nutrition habits according to disease severity.

Variables	Mild (*n* = 79)	Moderate to severe (*n* = 21)	*p*
Gender			
Male	40 (50.6%)	13 (61.9%)	0.5^a^
Female	39 (49.4%)	8 (38.1%)	
Age	29.5 ± 11.3	32.5 ± 12.3	0.17^b^
BMI	24.50 ± 4.15	27.39 ± 4.76	**0.018** ^c^
AFCH	9.01 ± 3.68	8.57 ± 4.06	0.46^b^
Diet preference			
Mostly meat	15 (19.0%)	2 (9.5%)	0.42^e^
Vegetarian	2 (2.5%)	0 (0%)	
Mixed	62 (78.5%)	9 (90.5%)	
Oil preference			
Vegetable oil	21 (26.6%)	4 (19.0%)	**0.008** ^d^
Animal oil	0 (0%)	2 (9.5%)	
Margarine	0 (0%)	1 (4.8%)	
Mixed	58 (73.4%)	4 (66.7%)	
Salt consumption			
Less salty	29 (36.7%)	2 (9.5%)	0.056^a^
More salty	15 (19.0%)	6 (28.6%)	
Normal	35 (44.3%)	13 (61.9%)	
Spice consumption			
Less spicy	24 (30.4%)	8 (38.1%)	0.45^a^
More spicy	13 (16.5%)	5 (23.8%)	
Normal	42 (53.2%)	8 (38.1%)	
Tea–coffee consumption			
1–4/day	39 (49.4%)	8 (38.1%)	0.76^d^
5–6/day	4 (5.1%)	2 (9.5%)	
7–10/day	11 (13.9%)	4 (19.0%)	
≥ 10/day	18 (22.8%)	6 (28.6%)	
Never	7 (8.9%)	1 (4.8%)	
Sugar consumption			
1/month	0 (0%)	1 (4.8%)	**0.050** ^d^
2/month	1 (1.3%)	0 (0%)	
1–2/week	15 (19.0%)	8 (38.1%)	
3–6/week	10 (12.7%)	3 (14.3%)	
Every day	29 (36.7%)	8 (38.1%)	
Never	24 (30.4%)	1 (4.8%)	
Bread consumption			
1/month	2 (2.5%)	1 (4.8%)	0.76^d^
2/month	1 (1.3%)	0 (0%)	
1–2/week	7 (8.9%)	1 (4.8%)	
3–6/week	14 (17.7%)	2 (9.5%)	
Every day	48 (45.6%)	10 (76.2%)	
Never	7 (8.9%)	1 (4.8%)	
Milk consumption			
1/month	7 (8.9%)	2 (9.5%)	0.12^d^
2/month	3 (3.8%)	3 (14.3%)	
1–2/week	34 (43.0%)	6 (28.6%)	
3–6/week	8 (10.1%)	5 (23.8%)	
Every day	12 (15.2%)	4 (19.0%)	
Never	15 (19.0%)	1 (4.8%)	
Vegetable–fruit consumption			
1–2/week	36 (32.9%)	6 (28.6%)	0.98^d^
1/day	26 (32.9%)	6 (28.6%)	
2/day	10 (12.7%)	3 (14.3%)	
≥ 3/day	7 (8.9%)	2 (9.5%)	
Never	0 (0%)	0 (0%)	
Red meat consumption			
1–2/week	67 (84.8%)	14 (66.7%)	0.25^d^
1/day	8 (10.1%)	4 (19.0%)	
2/day	2 (2.5%)	2 (9.5%)	
≥ 3/day	2 (2.5%)	1 (4.8%)	
Never	0 (0%)	0 (0%)	

*Note:* Bold value of *p* 〈 0.05 was considered statistically significant.

Abbreviations: AFCH, Adolescent Food Habits Checklist; BMI, body mass index.

^a^ Pearson chi‐Squared.

^b^ Mann–Whitney *U* test.

^c^ Independent student *t*–test.

^d^ Pearson exact chi‐squared.

^e^ Fisher exact chi‐squared.

### 3.2 Evaluation of Nutrition Habits

The AFHC score was 8.92 ± 3.75 in the patients with SD and 10.3 ± 3.97 in the healthy controls, and it was significantly lower in the patients with SD (*p* = 0.009). It was found that 2% (*n* = 2) of the patients with SD preferred a vegetarian diet, 17% (*n* = 17) preferred a meat‐based diet, and 81% (*n* = 81) preferred a mixed diet, whereas 0.9% (*n* = 1) of the control group preferred a vegetarian diet, 21.8% (*n* = 24) preferred a meat‐based diet, and 77.3% (*n* = 85) preferred a mixed diet (*p* = 0.56). In addition, fat, salt, spice, tea–coffee, sugar, and milk consumption was similar in the patients with SD and controls (*p* > 0.05). However, bread consumption was more frequent in the patients with SD (*p* = 0.001). (Table 1).

The mean AFHC score was 9.01 ± 3.68 in the mild disease group and 8.57 ± 4.06 in the moderate to severe patients with SD. The AFHC score was lower in the patients with moderate to severe SD, however it was not statistically significant (*p* = 0.462). Dietary preferences were similar in the mild and moderate to severe SD groups (*p* = 0.427). Moreover, salt, spice, tea, coffee, milk, and bread consumption was similar in the mild and moderate to severe SD groups (*p* > 0.05). Consumption of animal fat and margarine was more frequent in the moderate to severe patients whereas vegetable oil was more frequent in the mild patients (*p* = 0.008). In addition, sugar consumption was more frequent in the patients with moderate to severe SD (*p* = 0.050) (Table 2).

### 3.3 Evaluation of Psychoemotional Status

The mean depression, anxiety, and stress subscales of DASS‐21 and total DASS‐21 scores were higher in the patients with SD than the controls, however it was not statistically significant (*p* = 0.33, *p* = 0.089, *p* = 0.16, *p* = 0.16, respectively). However, the mean anxiety subscale and total DASS‐21 scores were significantly higher in the moderate to severe disease group than mild disease group (*p* = 0.035, *p* = 0.049, respectively). The mean depression and stress subscale scores were also higher in the moderate to severe group, but there was no statistically significant difference (*p* = 0.12, *p* = 0.15, respectively) (Table 3).

**TABLE 3:** Psychiatric scales according to disease severity.

Variables	Mild	Moderate to severe	*p*
DASS 21	13.8 ± 10.8	18.1 ± 10.5	**0.049**
	11.0 (6.00–17.5)	17.0 (8.00–26.0)	
Depression subscale	4.43 ± 4.51	5.86 ± 4.33	0.12
	3.00 (1.00–6.00)	6.00 (2.00–9.00)	
Anxiety subscale	3.91 ± 3.41	5.33 ± 3.09	**0.035**
	3.00 (1.00–5.50)	6.00 (3.00–8.00)	
Stress subscale	5.58 ± 4.09	6.90 ± 4.13	0.15
	5.00 (3.00–8.00)	7.00 (4.00–10.0)	

*Note:* Bold value of *p* 〈 0.05 was considered statistically significant.

Abbreviation: DASS‐21, Depression Anxiety Stress Scale‐21.

### 3.4 Correlation of SDASI and the Other Parametric Data

When the relationship between SDASI and age, BMI, AFHC score, total DASS‐21, and its subscales scores in patients with SD was evaluated, a similar relationship was found between SDASI and BMI (*p* = 0.018) (Table 4).

**TABLE 4:** Correlation of SDASI and the other parametric data.

	Age	BMI	AFCH	Depression	Anxiety	Stress	DASS‐21
**SDASI**	*r* = 0.054	*r* = 0.237	*r* = 0.041	*r* = 0.139	*r* = 0.152	*r* = 0.116	*r* = 0.157
	*p* = 0.594	***p* = 0.018**	*p* = 0.683	*p* = 0.166	*p* = 0.132	*p* = 0.250	*p* = 0.120

*Note:* Bold value of *p* 〈 0.05 was considered statistically significant.

Abbreviations: AFCH, Adolescent Food Habits Checklist; BMI, body mass index; DASS‐21, Depression Anxiety Stress Scale‐21; SDASI, Seborrheic Dermatitis Area Severity Index.

## 4 Discussion

There are studies evaluating the relationship between nutrition habits and various sebaceous gland diseases such as acne, rosacea, and androgenetic alopecia [8, 9, 10]. However, there are not much data on SD and nutrition. Genetic predisposition, detriment of barrier function, increased sebum secretion, and microecological disturbance are critical factors in the pathogenesis of SD [17]. The microbiome is very important for immune system and homeostasis. *Malassezia* spp. has a crucial role in the development of SD. Moreover, count of microorganisms such as *Staphylococcus, Candida, Aspergillus* and *Filobasidium* have also been shown to increase in individuals with SD [18]. Cutaneous flora is affected by a wide variety of gut microbiome [19]. Fecal microbiome profile has an important role on health and disease. The fecal microbiome can be positively or negatively affected according to nutrition habits [20].

Increased sebum secretion has a great importance in the pathogenesis of SD. Sebum maintains epidermal barrier function, transports antioxidants to the skin surface and protects the skin from microbial colonization [21]. Nutrition habits especially fat, sugar, and spicy food are thought to affect skin conditions [22]. In a study, it was reported that gastrointestinal dysfunction, consumption of sweet and spicy food is a significant risk factor for sebaceous gland diseases [23]. Diet has a significant impact on sebum. Dietary lipids (fatty acids), glucose, and acetate intake, which are important substrate sources for sebum synthesis, affect sebaceous gland activity [17, 24]. Diets rich in high glycemic index carbohydrates give rise to hyperglycemia, resulting in reactive hyperinsulinemia and increased insulin‐like growth factor‐1 (IGF‐1) [24]. In a study, it was shown that serum IGF‐1 and the amount of facial sebum were positively correlated [25]. Consuming carbohydrates with high glycemic index may contribute to the development or exacerbation of SD by stimulating sebum secretion [26]. In our study, bread consumption was more frequent in the patients with SD. Sugar consumption was also more frequent but not statistically significant, and there was no association between milk and fat consumption. However, sugar and unhealthy fat (animal fat, margarine) consumption were more frequent in the moderate to severe disease group. Similarly, Bett et al. [27] also showed that sugar consumption was more frequent in patients with SD.

Sanders et al. [5] showed that western dietary pattern was related to higher SD. However, in our study, dietary preference (vegetarian, meat‐based, mixed) was similar in patients with SD and controls. Tamer [26] reported that vegetable consumption was more frequent in the patients with SD. In contrast, Sanders et al. [5] reported that higher fruit in daily diet was related to lower SD. In our study, there was less frequent vegetable–fruit consumption in the patients with SD.

The presence of metabolic syndrome (MS) is thought to be a trigger for the development of skin diseases. Insulin resistance and visceral obesity may lead to MS by causing chronic inflammation. MS is associated with genetics and environmental factors like nutrition habits. Many cutaneous diseases such as psoriasis, acanthosis nigricans, and acne vulgaris have been associated with MS [28, 29]. Recent studies also showed that SD is associated with MS [29, 30]. One indicator of visceral obesity is BMI. BMI has been reported to increase the occurrence of SD [30]. Linder et al. [3] reported that obesity was more common in patients with SD than in the normal population. BMI was higher in the patients with SD, but it was not statistically significant in our study. However, it was higher in the patients with moderate to severe SD than in the patients with mild SD. Moreover, a positive correlation between disease severity and BMI was detected. Obesity may lead to the onset of SD or more severe SD through chronic inflammation.

AFHC is a scale which assesses healthy eating habits. A high score of AFHC means more healthy eating habits. It has been evaluated in sebaceous gland diseases such as acne and androgenetic alopecia [8, 10]. To our knowledge, AFHC has not been assessed in patients with SD. In our study, it was found that patients with SD had unhealthy eating habits similar to the patients with acne and androgenetic alopecia [8, 10]. Moreover, the AFHC score was lower in patients with moderate to severe SD than patients with mild SD, but this difference was not statistically significant. These data suggested that healthy nutrition habits are very important in SD as in other sebaceous gland diseases. Healthy nutrition habits may contribute to prevent both the occurrence and exacerbation of SD. They will also prevent comorbidities that may accompany SD, such as MS. Therefore, patients with SD should be encouraged to adopt healthy nutrition habits.

The pathophysiological link between stress factors and cutaneous diseases have not been fully understood. However, stress is thought to contribute to inflammation by regulating the hypothalamic–pituitary–adrenal axis and secreting neuropeptides, neurotrophins, lymphokines, and other chemical mediators from nerve endings and dermal cells [31]. SD may cause psychosocial effects, especially in the scalp and face involvement. Studies have shown that high stress levels are a risk factor for SD exacerbations [32, 33]. There is very few information regarding SD and psychoemotional status in the literature. In our study, anxiety subscale and total DASS‐21 score were significantly higher in the patients with moderate to severe SD. In a recent study, Cömert et al. [6] showed that anxiety scores were higher in patients with SD however, not related to severity of disease. Another study reported that stress is a triggering factor for SD episodes. It was also shown that depression score was not associated with episodes, but anxiety score was [34]. These data show that psychoemotional status particularly anxiety is substantial in patients with SD. High anxiety may accompany patients with SD and lead to severe disease. Therefore, psychoemotional status of patients should be assessed.

This study has some limitations. This is a single‐center prospective study and has relatively few patients and including only Turkish population. But this study is including diet and psychoemotional status in SD. These data are very few in literature. Despite these restrictions, we believe that the consequences are worthful.

In conclusion, there was a positive correlation between the severity of SD and BMI. The AFHC score was lower in patients with SD who consumed more bread and less fruits and vegetables. Margarine, animal fat, and sugar consumption was higher in patients with moderate to severe SD. DASS‐21 anxiety subscale and DASS‐21 total scores were higher in the moderate to severe group. This study suggests that nutrition habits, obesity, and psychoemotional status particularly anxiety may have a critical effect in the etiopathogenesis of SD. Healthy nutrition habits and psychoemotional status may prevent the occurrence and exacerbation of SD. We also think that they may be crucial in the management of concomitant comorbidities such as MS.

## Author Contributions

All authors read and accepted the last manuscript. T.B., E.A., H.K.E., E.A., M.B., and Z.N.S. designed the research study, T.B., E.A., H.K.E., E.A., M.B., and Z.N.S. performed the research, T.B., E.A., H.K.E., E.A., M.B., and Z.N.S. analyzed the data, and T.B. and E.A. wrote the paper.

## Ethics Statement

Eskişehir Osmangazi University, local ethics committee approved the study protocol (Decision no: 2020/16).

## Conflicts of Interest

The authors declare no conflicts of interest.

## Data Availability

The data that support the findings of this study are available from the corresponding author upon reasonable request.
